# Current News

**Published:** 1905-05

**Authors:** 


### Current News.

### NATIONAL ASSOCIATION OF DENTAL FACULTIES.

The annual meeting of the National Association of Dental Faculties will be held at Buffalo, commencing at two P.M. on Thursday, July 27, 1905. The Executive Committee will meet at ten A.M. same day. Special business to come before the National Association of Dental Faculties is the consideration of the proposed revision of the Constitution and By-Laws.

H. B. Tileston,
*Chairman Executive Committee.*
John I. Hart,
*Secretary Executive Committee.*

### NATIONAL DENTAL ASSOCIATION.

The ninth annual session of the National Dental Association will be held in Buffalo, N. Y., July 25 to 28, 1905, inclusive.

The Hotel Iroquois has been selected by the Local Committee of Arrangements as head-quarters, where all general sessions of the Association and of the sections will be held. The clinics will be held at the rooms of the Dental Department, University of Buffalo.

Rates at the Hotel Iroquois are: Single room, per day, $1.50, $2.00, and $2.50; rooms for two persons, $3.00 and $4.00; single rooms with bath, $3.00 and $3.50; rooms with bath for two persons, $5.00, $6.00, $7.00, and $7.50; all rooms on the European plan.

The usual railroad rate of one and one-third fare for the round trip, certificate plan, has been arranged for by the Executive Committee.

All pay full fare going, taking the proper certificate therefor from the ticket agent, which, when properly viséd at the meeting, entitles the holder to return for one-third the regular rate.

Tickets going may be purchased from July 20 to 26, and are good returning to and including August 2.

Both the general officers and those of the sections have been working hard to provide an interesting and instructive programme, and a large attendance is expected.

A. H. Peck,
*Recording Secretary.*

92 State Street, Chicago, Ill.

---

### NATIONAL DENTAL ASSOCIATION CLINIC.

The National Dental Association will meet at Buffalo, N. Y., commencing July 25, 1905. It is the desire of the president and chairman of the Clinic Section to hold the best clinic in the history of the society. The clinics will be held Wednesday and Thursday, July 26 and 27, in the Buffalo Dental College, where there is every facility for making practical operations, as well as ample room for all those wishing to give table clinics. Forty dental operations will be made each day, and there is room for holding three hundred table clinics. Those interested can apply to

Dr. S. W. Bowles, 1315 New York Avenue, Washington, Chairman for District of Columbia, Delaware, and New Jersey.

Dr. E. C. Blasdell, 1 Pleasant Street, Portsmouth, N. H., Chairman for Maine, New Hampshire, and Vermont.

Dr. F. W. Gethro, 31 Washington Street, Chicago, Chairman for Illinois and Wisconsin.

Dr. L. L. Barber, Spitzer Building, Toledo, Ohio, Chairman for Ohio and Indiana.

Dr. S. Eschelman, 421 Franklin Street, Buffalo, N. Y., or Dr. R. Murray, 715 Elmwood Avenue, Buffalo, N. Y., Chairmen for New York and Ontario, Canada.

Dr. M. F. Finley, 1928 First Street, Washington, D. C., Chairman for District of Columbia, Virginia, and West Virginia.

Dr. T. P. Hinman, 22 South Broad Street, Atlanta, Ga., Chairman for Georgia, North Carolina, South Carolina, Florida, Alabama, Mississippi, Tennessee, Louisiana, and Texas.

Dr. H. B. McFadden, 3505 Hamilton Street, Philadelphia, Pa., Chairman for Pennsylvania.

Dr. G. E. Savage, 518 Main Street, Worcester, Mass., Chairman for Massachusetts, Connecticut, and Rhode Island.

Dr. S. H. Voyles, 3201 Washington Avenue, St. Louis, Mo., Chairman for Missouri, Arkansas, Kansas, and Nebraska.

Those having new instruments, appliances, etc., are cordially invited to display them. Communicate with your State Chairman, or with

E. K. Wedelstaedt,
*Secretary Clinic Section.*

204 New York Life Building, St. Paul, Minn.

---

### AMERICAN MEDICAL ASSOCIATION, SECTION ON STOMATOLOGY.

The next meeting of the American Medical Association will be held in Portland, Ore., July 11 to 14, 1905. The programme for the Section on Stomatology is as follows:

1. Chairman's Address. Vida A. Latham, Chicago.
2. " The Causes and the Treatment of the Mouth Manifestations of Certain Metabolic Disorders." Alfred C. Croftan, Chicago.
3. " The Oral Manifestations of Diabetes Mellitus." Herman Prinz, St. Louis, Mo.
4. " The Urine and Saliva in So-called Pyorrhœa Alveolaris." Wm. J. Lederer, New York City.
5. " Further Researches in the Treatment of Interstitial Gingivitis." Eugene S. Talbot, Chicago.
6. " Excretion of Toxic Products into the Mouth, with Relation to Local Infection." Fenton B. Turck, Chicago.
7. " The Relations of Dentistry to General Medicine." Samuel Hopkins, Boston, Mass.
8. " A Common Ground for Medicine and Dentistry." Frank L. Platt, San Francisco.
9. " The Physician as a Dentist." Calvin W. Knowles, San Francisco.
10. " The Physician's Duty to the Child from a Dental Stand-Point." Alice M. Steeves, Boston, Mass.
11. " Dentistry of To-morrow." H. P. Carlton, San Francisco.

12. "What will probably be the Dental Educational Standard for the Coming Decade?" C. C. Chittenden, Madison, Wis.

13. "Fatal Oral Pathologic Conditions." G. V. I. Brown, Milwaukee, Wis.

14. "Surgical Bacteriology of the Mouth." A. H. Levings, Milwaukee, Wis.

15. "Surgical Aspects of Disturbed Dentition of the Third Molar." M. L. Rhein, New York City, N. Y.

16. "The Treatment of Suppurative Affections of the Face and Neck emanating from the Mouth." M. I. Schamberg, Philadelphia.

17. "The Medical Relations of Certain Conditions of the Mouth." L. Duncan Bulkley, New York City.

18. "Some Effects of Inebriety on the Teeth and Jaws." T. D. Crothers, Hartford, Conn.

19. "The Ossification of the Lower Jaw." Edward Fawcett, Bristol, England.

20. "Ankylostomiasis and Tongue Pigment." T. M. Russell Leonard, Grenada, British West India.

21. "Notes on Tooth Genesis in Man." H. W. Marett Tims, London, England.

22. "The Etiology of Tooth Corrugations." G. Lenox Curtis, New York City.

23. "To what Extent are Teeth necessary to the Human Being?" M. H. Fletcher, Cincinnati, Ohio.

24. "Anæsthesia by Ethyl Chloride and Similar Agents." H. C. Miller, Portland, Ore.

25. "The Röntgen Rays in Dentistry." M. Kassabian, Philadelphia.

The programme is entirely scientific. All dentists are invited to be present and take part in the discussions. Those wishing to become members may do so by filling out blanks furnished by the Association, signed by the President and Secretary of State or local dental or medical society, enclosing five dollars, and sending to the Secretary of the Section on Stomatology for his signature. This also includes the *Journal of the American Medical Association* for one year.

VIDA A. LATHAM, *Chairman.*
EUGENE S. TALBOT, *Secretary.*

### NEW JERSEY STATE DENTAL SOCIETY.

The thirty-fifth annual meeting of the New Jersey State Dental Society will be held in the Auditorium, Asbury Park, N. J., commencing July 19, and continuing until July 22, 1905. Headquarters at Hotel Columbia. Rates for one person in room, $3.50; two persons in room, $3.00. Meeting commences promptly at ten A.M. on the 19th. The various committees have been successful in securing eminent practitioners for papers of present interest. Some fifty clinicians in the most modern up-to-date dentistry, and the space in the large Auditorium almost entirely filled with all the newest appliances to practise dentistry.

Friday evening will be devoted to the social side, with a smoker, including a collation and entertainment to the guests, exhibitors, and members. Cut out now the week of July 17, and meet with us.

Seven hundred and fifty-six dentists were registered last July. Make it a thousand this year.

CHARLES A. MEEKER,
*Secretary.*

---

### MINNESOTA STATE BOARD OF DENTAL EXAMINERS.

The Minnesota State Board of Dental Examiners will hold a special examination, June 5, 6, and 7, 1905, at the Dental Department of the State University.

The Secretary will be at the Dental Department on the afternoon of June 3 to receive applications. All applications must be in by five P.M. of that date. Application blanks will be furnished upon request, by the Secretary.

F. S. JAMES,
WINONA, MINN. *Secretary.*

---

### NORTHERN OHIO DENTAL ASSOCIATION.

The forty-sixth annual meeting of the Northern Ohio Dental Association will be held June 6, 7, and 8, 1905, at Gray's Armory, Cleveland, Ohio.

This is not only one of the oldest, but is one of the best-attended, meetings in the country. This year the programme is one of unusual strength and interest. The leading subjects for consideration are—

1. Humanitarian Methods.
2. Mistakes.
3. Prophylaxis.

Under the first is considered "High Pressure Anæsthesia," by Dr. C. G. Myers, of Cleveland; and "High Pressure Anæsthesia as compared with other Pain Preventing Methods," by Dr. D. H. Zeigler, of Cleveland.

Essays under the second group include "Mistakes of the Country Dentists," by Dr. R. D. Wallace, Scio, Ohio, "Mistakes of the City Dentists," by Dr. F. J. Spargur, Cleveland, Ohio, and "Mistakes in Ethics," by Professor S. H. Guilford, of Philadelphia, Pa.

The third includes the Essays, "Two Sources of Tooth Life and their Relative Importance," by Dr. D. D. Smith, of Philadelphia, Pa., and "Diseases of the Peridental Membrane and Treatment," by Dr. J. V. Stahl, of Wooster, Ohio.

The Essayists and those who open discussion upon the various papers have been selected for their particular fitness to handle subjects assigned to them.

Under "Mistakes in Ethics," Dr. Guilford will point out, as only he can, some mistakes that are being made by the profession in the relation of its members to each other, together with the mistakes made in treatment of patients and the public. Great good is expected to result from the presentation of this paper and the discussions that follow. Many false impressions have existed in the past and still exist as to the duties we owe to each other, our patients, and the public, and it is expected that the three papers on mistakes will do much to correct this.

Dr. Smith's paper bears upon that all important subject, prophylaxis. He will bring with him a patient showing results accomplished by his method of procedure. He will illuminate his paper with models and instruments.

Throughout the entire programme much attention will be given to the study of Humanitarian Methods (methods which make it possible to perform dental operations free from pain).

The two papers, "Application of High Pressure Anæsthesia," and "High Pressure Anæsthesia as compared with other Pain Preventing Methods," and the discussions to follow, will set forth all that is known of importance in this connection.

There will be about fifty clinics selected and arranged to give the knowledge-seeking dentists the best post-graduate course that can possibly be obtained in a three days' meeting. One session will be devoted to the study of manufacturers' exhibits. The exhibits this year are to be one of the interesting features of the meeting, and the committee has been promised one of the largest exhibits shown in the country.

All communications pertaining to clinics or exhibits should be addressed to the Corresponding Secretary, Dr. W. G. Ebersole, 800 Schofield Building, Cleveland, Ohio.

Special rate of a fare and a third have been granted on the certificate plan by the Central Passenger Association.

The Committee extend a most cordial invitation to the members of the profession to attend.

W. G. Ebersole,
Geo. H. Wilson,
Varney E. Barnes,
*Executive Committee.*

---

### MISSOURI DENTAL ASSOCIATION.

The Missouri State Dental Association will meet in the city of St. Louis, May 24, 25, and 26, 1905.

An excellent programme of papers and clinics is being prepared, and all ethical members of the profession are cordially invited to attend.

Sam T. Bassett,
*Corresponding Secretary.*

---

### OKLAHOMA DENTAL ASSOCIATION.

The fifteenth annual meeting of the Oklahoma Dental Association will be held in Oklahoma City, commencing at eight P.M., May 15, and continuing until the evening of the 17th. Indications are for a large and enthusiastic meeting. The profession is cordially invited to attend.

C. L. White, D.D.S.,
Oklahoma City, Okla. *Secretary.*

### LEBANON VALLEY DENTAL ASSOCIATION.

THE thirtieth annual meeting of the Lebanon Valley Dental Association will be held at Pottstown, Pa., May 16 and 17, 1905.

Meetings, clinics, and exhibits will be held in the "Auditorium," a commodious and well-lighted hall, giving excellent opportunity for exhibits. For space, write the chairman of the Executive Committee,

C. R. SCHOLL.

READING, PA.

---

### SOUTH DAKOTA STATE BOARD OF DENTAL EXAMINERS.

THE next meeting of the South Dakota State Board of Dental Examiners will be held at Mitchell, S. D., July 11, beginning at 1.30 P.M. All candidates will be required to do practical work in both operative and prosthetic dentistry, and should bring all instruments and materials necessary to do the same. Vulcanizer, lathe, and swaging appliances will be furnished by the board. Application, together with fee of ten dollars must positively be in the hands of the Secretary before July 7.

G. W. COLLINS.

---

### "F. D. I." INTERNATIONAL DENTAL FEDERATION.

THE next annual meeting of the Executive Council of the Fédération Dentaire Internationale will convene in Hanover, Germany, August 7, 1905, immediately following the annual meeting of the Central-Verein Deutscher Zahnarzte. Announcement of the programme for the meeting and the projected work for the Federation during the present period will shortly be made through the dental journals and through the official bulletin of the Federation.

EDWARD C. KIRK,
*Secretary-General.*

